# Acute Effects of Glucose and Fructose Administration on the Neural Correlates of Cognitive Functioning in Healthy Subjects: A Pilot Study

**DOI:** 10.3389/fpsyt.2018.00071

**Published:** 2018-03-12

**Authors:** Davide Zanchi, Anne Christin Meyer-Gerspach, André Schmidt, Claudia Suenderhauf, Antoinette Depoorter, Jürgen Drewe, Christoph Beglinger, Bettina Karin Wölnerhanssen, Stefan Borgwardt

**Affiliations:** ^1^Department of Psychiatry (UPK), University of Basel, Basel, Switzerland; ^2^Department of Research, St. Clara Hospital, Basel, Switzerland; ^3^Division of Neuropediatrics and Developmental Medicine, University Children’s Hospital, Basel, Switzerland; ^4^Department of Biomedicine, University Hospital Basel, Basel, Switzerland

**Keywords:** functional magnetic resonance imaging, glucose, fructose, brain–gut, working memory, cognition

## Abstract

The present randomized double-blinded cross-over study aims to extensively study the neural correlates underpinning cognitive functions in healthy subjects after acute glucose and fructose administration, using an integrative multimodal neuroimaging approach. Five minutes after glucose, fructose, or placebo administration through a nasogastric tube, 12 participants underwent 3 complementary neuroimaging techniques: 2 task-based functional magnetic resonance imaging (fMRI) sequences to assess working memory (N-back) and response inhibition (Go/No-Go) and one resting state fMRI sequence to address the cognition-related fronto-parietal network (FPN) and salience network (SN). During working memory processing, glucose intake decreased activation in the anterior cingulate cortex (ACC) relative to placebo, while fructose decreased activation in the ACC and sensory cortex relative to placebo and glucose. During response inhibition, glucose and fructose decreased activation in the ACC, insula and visual cortex relative to placebo. Resting state fMRI indicated increased global connectivity strength of the FPN and the SN during glucose and fructose intake. The results demonstrate that glucose and fructose lead to partially different partially overlapping changes in regional brain activities that underpin cognitive performance in different tasks.

## Introduction

The mammalian brain depends upon sugars as the main source of energy, and the regulation of sugar metabolism is critical for brain physiology (1). Glucose and fructose, two of the most important monosaccharides, have a roughly equal number of calories but are metabolized differently (2). Glucose, a highly potent secretagogue, leads to the release of insulin and satiation hormones such as GLP-1 by enteroendocrine cells as well as inhibition of the appetite inducer ghrelin (3, 4). In contrast, fructose intake does not affect the release of insulin to the same extent (5, 6) and chronic fructose consumption may adversely affect human health by leading to increased *de novo* lipogenesis in the liver, hyperuricemia, and obesity (7, 8).

The differences in the metabolism of glucose and fructose may also explain their differential effects on neuronal pathways. Page’s milestone study (9) has documented reduced relative cerebral blood flow and increased functional connectivity after the ingestion of sugars (both glucose and fructose) in the insula, anterior cingulate, striatum, and posterior cingulate cortex (appetite and food-reward regions). The effects from fructose were greater, and this resulted in increased brain activation in the visual cortex during a food-cue task (2). Similar results were found by a recent study conducted by our group investigating resting state functional connectivity in the basal ganglia network (4).

Whereas changes linked to appetite stimulation in the human brain are generally accepted (2, 9, 10), recent animal studies suggest that sugars may have different effects on brain regional activity underlying cognitive functioning (11–14). While extensive evidence indicates that increased glucose concentrations enhance learning and memory processes in rodents through the enhancement of hippocampal activity (15), recent studies indicate that the hippocampus may be particularly vulnerable to the effects of fructose, with impaired synaptic plasticity and consequent decreased working memory performance after high-fructose diets (16, 17). To our knowledge, no previous studies have investigated the effects of glucose and fructose on whole-brain activity during different cognitive functions in humans.

Therefore, while dietary energy intake, in particular the consumption of simple sugars such as fructose, has been increasing steadily in Western societies, the effects of such a diet on the human brain are still poorly understood (17). In particular, food intake (as sugars) can have a significant role beside age and gender in multimodal neuroimaging studies (18). The present study can be considered as a starting point for future investigations also outside the brain-gut field, suggesting to assess nutrients intake in the functional magnetic resonance imaging (fMRI) analyses.

In the present study, we employ a functional multimodal approach to study the effects of glucose and fructose on different cognitive functions. We administered glucose and fructose to the participants through a nasogastric tube inserted into the stomach. After 5 min, the participants underwent an extensive fMRI examination, performing one N-back task (to assess working memory), one Go/No-go task (to assess response inhibition), and one resting state sequence focusing on two cognition-related resting state networks, in particular the fronto-parietal network (FPN) and salience network (SN).

As glucose and fructose are subject to differential metabolic processes at the cellular level (2), we hypothesized that these monosaccharides would also induce dissociable effects on brain regional activity during cognitive functioning.

## Materials and Methods

### Participants

The protocol was approved by the Ethics Committee of Basel, Switzerland (EKBB: 08/11) and conducted in accordance with the principles of the Declaration of Helsinki. All experimental procedures were carried out in accordance with the approved guidelines. The participants and the experiment protocol for the present study were already presented in a previous work of the same team (4). Fourteen (14) subjects were recruited through local and internet advertising. Each participant underwent a medical interview and laboratory screening and gave written informed consent prior to inclusion. Exclusion criteria were: lactose intolerance, smoking, substance abuse, regular intake of medications, medical or psychiatric illness, and any contraindication to MRI (e.g., claustrophobia, non-removable metal devices) or abnormalities detected upon laboratory screening. Of the 14 (14) subjects originally recruited, 2 had to be excluded as they did not meet the eligibility criteria. There was also one drop-out, who was replaced. The final sample included 12 healthy volunteers (mean age: 24.8 years, range: 21–31 years, and mean BMI: 22.9 kg/m^2^, range: 21.0–24.0 kg/m^2^).

### Experimental Protocol

This was a randomized, double-blind, cross-over study and was carried out at the Phase I Research Unit of the University Hospital of Basel. Glucose, fructose, and a placebo were administered to each subject on three different days, following the procedure described below. The treatment order was randomized and at least 7 days passed between the visits.

After an overnight fast of at least 10 h, an 8 F polyvinyl nasogastric tube was inserted into the subjects’ stomach through an anesthetized nostril and its intragastric position was verified by rapid injection of 10 ml air and auscultation of the upper abdomen.

The solutions were freshly prepared and were at room temperature when administered. Glucose monohydrate and fructose were purchased from Hänseler AG (Herisau, Switzerland). Different persons prepared and administered the solutions. Over 2 min, subjects received 300 ml of tap water with 75 g of glucose or with 25 g of fructose, or 300 ml pure tap water (placebo) *via* the nasogastric tube while sitting in the MR room. The administered doses were chosen on the basis of previous studies demonstrating lipogenesis increased in proportion after sugar intake (19).

Directly after administration, the tube was removed. To evaluate the treatment effect, the subjects underwent a brain imaging examination, including: three echo planar imaging (EPI) sequences (N-back task, Go/No-go task, and resting state sequence) and one T_1_ sequence.

### fMRI Acquisition

Scanning was performed on a 3T scanner (Siemens Magnetom Verio). The N-back task sequence was performed using an EPI sequence (TR = 2,500 ms, TE = 28 ms, flip angle = 83°, field of view = 228 mm × 228 mm, 32 slices, slice thickness: 3 mm; voxel size = 3.6 mm × 3.6 mm × 3.3 mm). In total, 126 EPI volumes were acquired. The Go/No-Go task sequence was performed using an EPI sequence (TR = 2,500 ms, TE = 28 ms, flip angle = 83°, field of view = 228 mm × 228 mm, 32 slices, slice thickness: 3 mm; voxel size = 3.6 mm × 3.6 mm × 3.3 mm). In total, 160 EPI volumes were acquired. The resting state EPI sequence had the following parameters: TR = 2,000 ms, TE = 28 ms, flip angle = 82°, field of view = 228 mm × 228 mm, 32 slices, slice thickness: 3.3 mm; voxel size = 3.6 mm × 3.6 mm × 3.3 mm. In total, 152 EPI volumes were acquired. Finally, the 3D T_1_-weighted structural scan had the following parameters: 256 × 256 matrix size, 176 sections, 1 mm × 1 mm × 1 mm TE = 3.37 ms, TR = 2,000 ms.

### N-Back Task

During the N-back task (20–22), all participants saw series of letters with an interstimulus interval (ISI) of 2 s. Each stimulus was shown for 1 s. During a baseline (0-back) condition, participants were required to press the button with the right hand when the letter “X” appeared. During 1-back and 2-back conditions, participants were instructed to press the button if the currently presented letter was the same as that presented in one (1-back condition) or two trials previously (2-back condition). The three conditions were presented in 10 alternating 30-s blocks (2 × 1-back, 3 × 2-back, and 5 × 0-back), matched for the number of target letters per block (i.e., 2 or 3), in a pseudorandom order. Task performance was expressed by the accuracy (number of correct responses to the 2-back task). A repeated measure of analysis of variance (ANOVA) was performed across the three visits.

### Go/No-Go Task

After the N-back task, all patients immediately underwent an event-related Go/No-Go fMRI paradigm that was conducted with jittered ISIs and containing infrequently presented oddball stimuli to optimize statistical efficiency. This is a well-validated paradigm (23, 24), requiring either the execution or the inhibition of a motor response, depending on the visual presentation of the stimuli. The basic Go task is a choice reaction time paradigm, in which arrows point either to the left or to the right for 500 ms, with a mean ISI of 1,800 ms (jitter range: 1,600–2,000 ms). During Go trials, subjects were instructed to press the left or the right response button according to the direction of the arrow. In 11% of the trials, arrows pointing upward appeared. During these so-called “No–Go” trials, participants were required to inhibit their motor response. During another 11% of the trials, arrows pointing left or right at a 23° angle were shown, and the subjects were told to respond in the same way as to Go stimuli (even though they pointed obliquely). These “oddball” stimuli were used as a control of the novelty effects associated with the low frequency and different orientation of the No–Go relative to the Go trials (stimulus-driven attention allocation). In total, there were 24 No–Go, 160 Go, and 24 oddball trials, with task durations of approximately 6 min.

### Statistical Analysis Software

The statistical analyses were conducted using GraphPad Prism (Version 6, GraphPad Software, San Diego, CA, USA) and FSL (Version 5.0.9, FMRIB, Oxford, UK).

### Analysis of Cognitive Performance

#### N-Back Task

To compare the performance during the N-back task, the reaction time and the number of correct answers (accuracy) were investigated for all conditions. A repeated measure ANOVA was performed with Tukey correction for *post hoc* pair-wise comparisons.

#### Go/No-Go Task

To compare the performance during the Go/No-go task, the reaction time and the “probability of inhibition” (ratio between No-Go correct and incorrect response) were investigated for all conditions. A repeated measure ANOVA was performed with Tukey correction for *post hoc* pair-wise comparisons.

### Task-Based Functional Imaging Analyses

#### Pre-Processing

Processing and analysis of imaging data were performed using FSL FEAT (fMRI Expert Analysis Tool version 6.00, http://fsl.fmrib.ox.ac.uk/fsl/fslwiki/FEAT). Pre-processing included brain extraction using FSL’s brain extraction tool, motion correction using FSL’s MCFLIRT (intra-modal motion correction tool) (25) and smoothing using FSL’s SUSAN (noise reduction uses non-linear filtering) (26). Images were finally normalized to MNI space.

#### N-Back Task

After pre-processing, the linear-model analysis of the N-back sequence included two levels. At the first level, the contrast “2-back vs. 0-back” was calculated separately for each participant. At the second level, group differences between glucose, fructose, and placebo were investigated. This resulted in a mixed-effects group model implementing FLAME 1 (FMRIB’s Local Analysis of Mixed Effects). Finally, a repeated measures permutation-based non-parametric test (randomize, FSL tool) was applied, correcting for multiple comparisons by threshold-free cluster enhancement (27). *p*-Values <0.05 were considered as significant.

#### Go/No-Go Task

After pre-processing, general linear models (GLM) analysis were performed to investigate brain activation differences during the Go/No-go sequence. At the first level, the contrast “No-go vs. oddball” was calculated separately for each participant. At the second level, group differences between glucose, fructose, and placebo were investigated. As above, a repeated measures permutation-based non-parametric approach (randomized, FSL tool) was applied, correcting for multiple comparisons by threshold-free cluster enhancement (27). *p*-Values <0.05 were considered as significant.

### Functional Resting State Connectivity Analysis

#### Resting State Network Identification

After pre-processing, to define brain networks at rest, an independent component analysis (ICA) was carried out on the resting state data using FSL’s multi-session multivariate exploratory linear optimized partition into independent components (MELODIC multi-session temporal concatenation) (28), setting the number of components to 20, which is common practice in ICA for fMRI data. Out of these 20 components, we decided to select and focus our analyses on 2 resting state networks (RSN): the fronto-parietal (also called executive functions) (FPN) network and the SN—identified as consistent with our previous studies (29, 30)—due to their involvement in cognitive functions and cognitive control (31–33). Cross-correlation of the two time-series, timepoint by timepoint, using as reference RS maps of Laird (30) were performed to compare the EF and SN networks to a major RSN template using a higher number of subjects.

#### RSNs Group Comparison

A dual regression approach (34) was carried out on the resting state data within the boundaries of the identified RSN. Region-averaged time courses of each subject for the three resting state networks were extracted and submitted to a repeated measure ANOVA to test for differences between the treatments, using Tukey correction for *post hoc* pair-wise comparisons.

### Cross-Modalities Correlations

After the task-based and resting state studies, we performed cross-modalities correlations analyses.

The regional averaged time-course was extracted across the subjects for the N-back and the Go/No-go for the significant contrasts. Moreover, connectivity values from the identified component for the resting state analyses were used. Individual correlation analyses across modalities were performed. FDR multiple comparisons corrections were used (Table 1).

**Table 1:** Cross-modality correlations.

	N-back	Go/No-go	FPN	SN
N-back		0.048	−0.316*	−0.341*
Go/No-go	0.048		−0.465*	−0.333*
FPN	−0.316*	−0.465*		−0.383*
SN	−0.341*	−0.333*	−0.383*	

After performing task-based and resting state analyses, we carried out cross-modalities correlations analyses. Significant correlations were found between the N-back results and resting state connectivity strength, both for the FPN (*p* < 0.05) and salience network (SN) (*p* < 0.05). For the SN, significant correlations were found between the Go/No-go results and the resting state connectivity values (*p* < 0.05). The results are corrected for FDR multiple comparison corrections. Pearson *R* values are displayed. Significant levels are reported using the conventional*.

## Results

### Behavioral Results

#### N-Back Task

The ANOVA showed no significant differences in accuracy across treatments.

#### Go/No-Go Task

No significant treatment differences were found for the probability of inhibition.

### N-Back Activations

Absolute values for motion are (mean ± SD): glucose 0.14 ± 0.04, fructose 0.17 ± 0.05, placebo 0.12 ± 0.04. Relative values for motion are (mean ± SD): glucose 0.04 ± 0.01, fructose 0.046 ± 0.01, placebo 0.042 ± 0.01.

**Figure 1:**
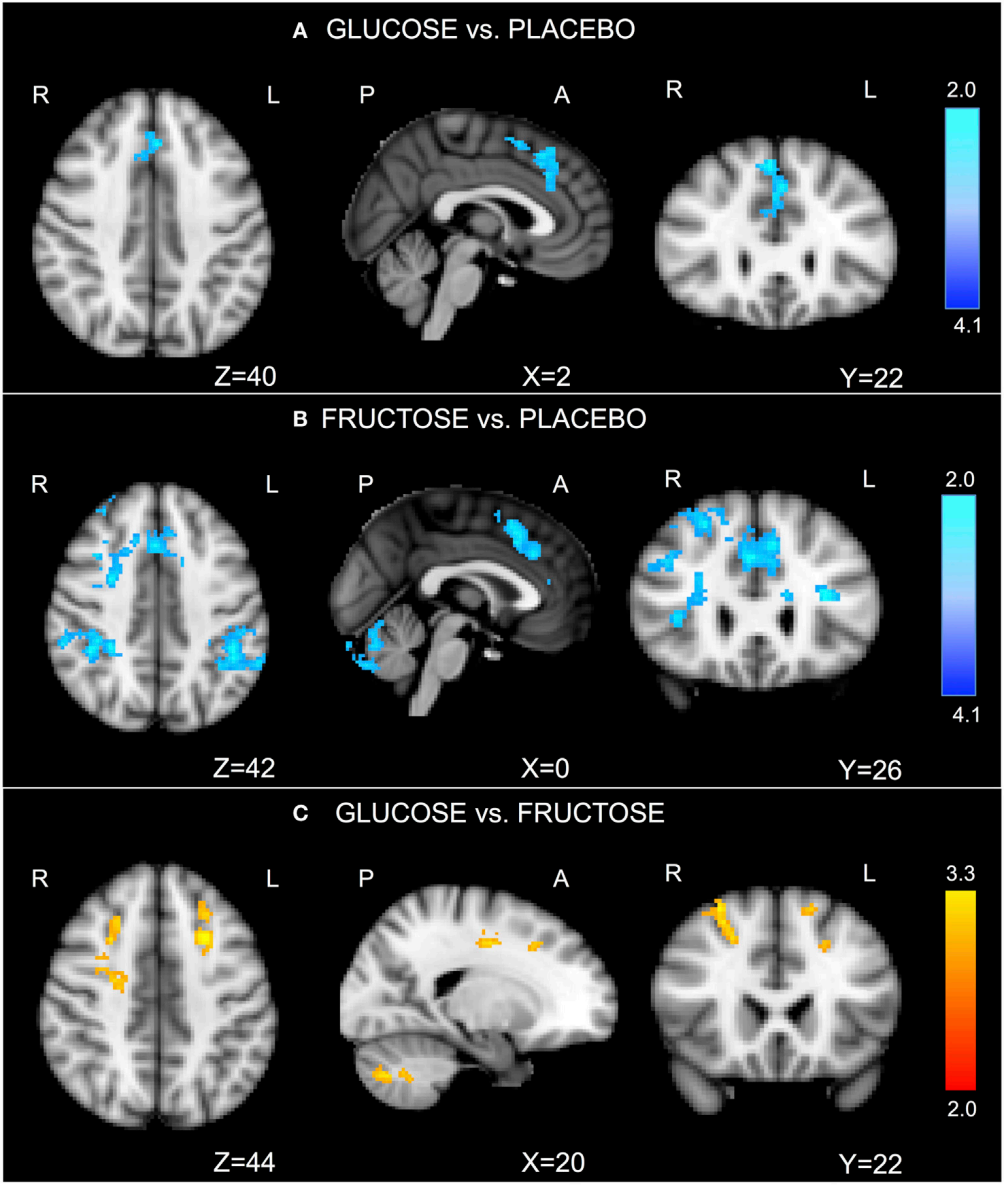
N-back functional imaging results. In the task-related general linear models, we considered the contrast of “2-back versus 0-back.” The comparison “glucose vs. placebo” revealed significantly reduced activation after ingesting glucose in the anterior cingulate cortex (ACC)/dorsal pre-frontal cortex [**(A)**, Table S1A in Supplementary Material]. The comparison “fructose vs. placebo” revealed significantly lower activations after ingesting fructose, particularly in the ACC/dorsal pre-frontal cortex, sensory cortex, and cerebellum [**(B)**, Table S1B in Supplementary Material]. The comparison “fructose vs. glucose” revealed significantly greater activations after ingesting fructose in the bilateral dorsal pre-frontal cortex and cerebellum [**(C)**, Table S1C in Supplementary Material]. *Z*-stat values are shown in the color bar. The results are given by repeated measures permutation-based non-parametric test (randomize, FSL tool) approach, correcting for multiple comparisons by threshold-free cluster enhancement (27). *p*-Values <0.05 were considered as significant.

In the task-related GLM, we considered the contrast of “2-back versus 0-back.” Relative to placebo, glucose significantly reduced activation in the anterior cingulate cortex (ACC)/dorsal pre-frontal cortex (Figure 1A; Table S1A in Supplementary Material). Relative to placebo, fructose significantly reduced activation in the ACC/dorsal pre-frontal cortex, sensory cortex, and cerebellum (Figure 1B; Table S1B in Supplementary Material). Glucose compared with fructose also significantly increased activation in the bilateral dorsal pre-frontal cortex and cerebellum (Figure 1C; Table S1C in Supplementary Material).

### Go/No-Go Activations

Absolute values for motion are (mean ± SD): glucose 0.16 ± 0.06, fructose 0.21 ± 0.07, placebo 0.17 ± 0.07. Relative values for motion are (mean ± SD): glucose 0.04 ± 0.01, fructose 0.04 ± 0.01, placebo 0.04 ± 0.01.

**Figure 2:**
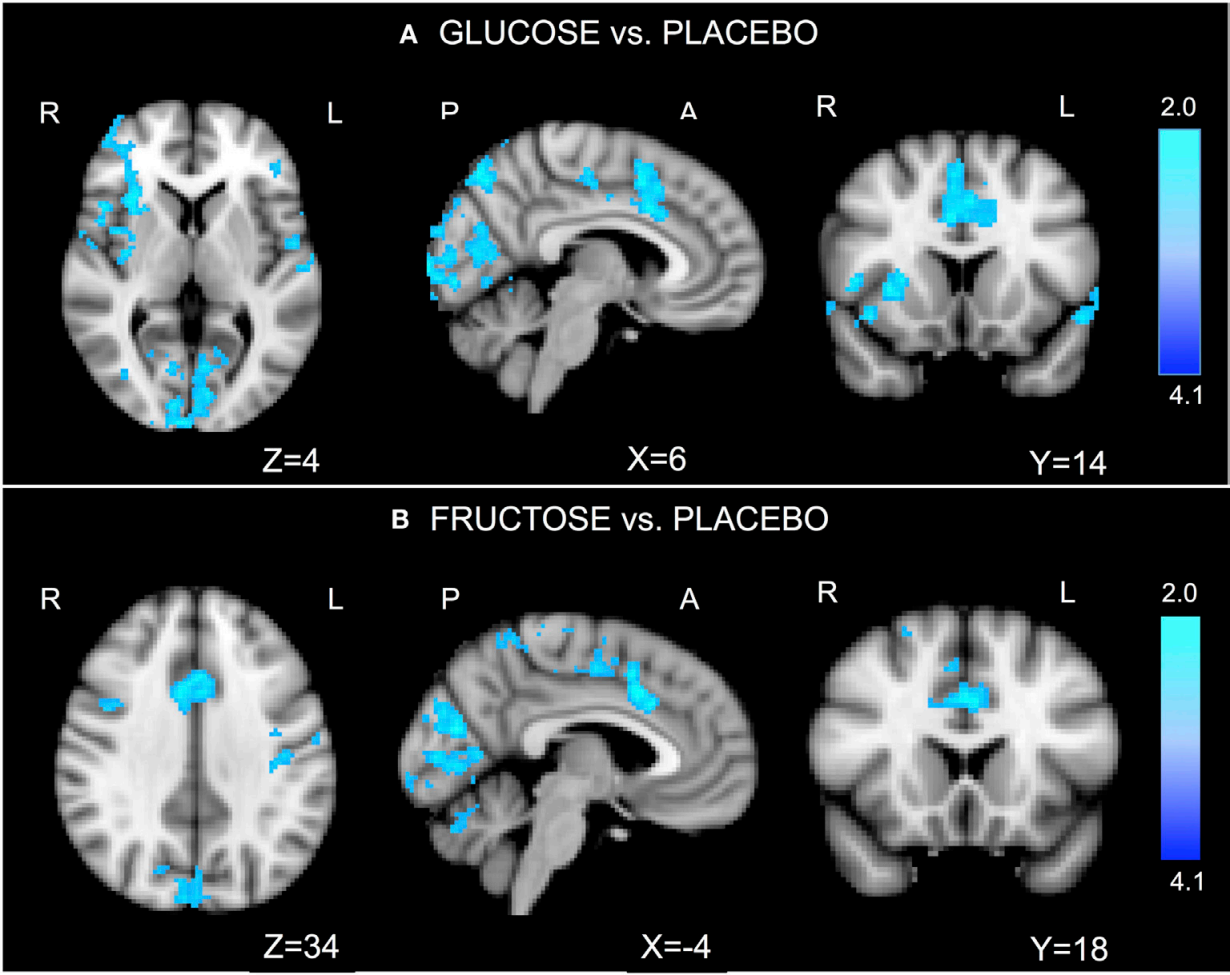
Go/No-go functional imaging results. In the task-related general linear models, we considered the contrast of “No-go versus Oddball.” The comparison “glucose vs. placebo” revealed significantly reduced activations after ingesting glucose, particularly in the anterior cingulate cortex (ACC), the dorsal pre-frontal cortex, right insula, and visual cortex **(A)**. The comparison “fructose vs. placebo” revealed significantly lower activations after ingesting fructose in the ACC, the dorsal pre-frontal cortex, sensor cortex, and visual cortex **(B)**. No significant differences were found for the comparison glucose vs. fructose. *Z*-stat values are shown in the color bar. The results are given by repeated measures permutation-based non-parametric test (randomize, FSL tool) approach, correcting for multiple comparisons by threshold-free cluster enhancement (27). *p*-Values <0.05 were considered as significant.

Relative to placebo, glucose significantly reduced activation in the ACC, dorsal pre-frontal cortex, right insula, and visual cortex (Figure 2A; Table S2A in Supplementary Material). Relative to placebo, fructose significantly reduced activation in the ACC, dorsal pre-frontal cortex, sensory cortex, and visual cortex (Figure 2B; Table S2B in Supplementary Material). No significant differences were found between glucose and fructose.

### Functional Resting State Connectivity Analysis Results

Absolute values for motion are (mean ± SD): glucose 0.14 ± 0.03, fructose 0.17 ± 0.05, placebo 0.15 ± 0.04. Relative values for motion are (mean ± SD): glucose 0.06 ± 0.02, fructose 0.07 ± 0.02, placebo 0.06 ± 0.02.

**Figure 3:**
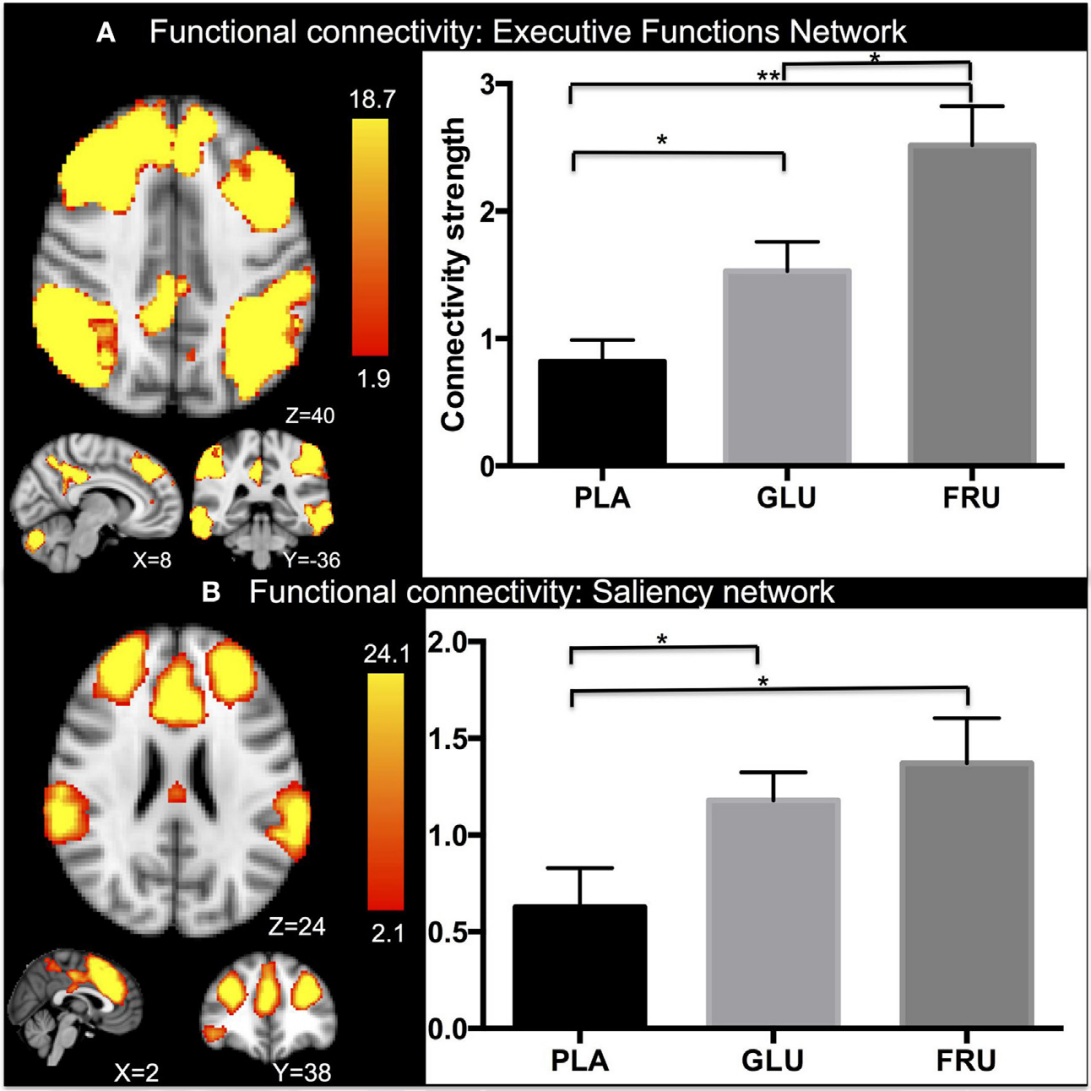
Independent component analyses results. After dual regression on the executive functions network (EF) and extracting the connectivity strength values, repeated measure analysis of variance (ANOVA) revealed significant activation in the EC network for the three groups **(A)**. In particular, significantly higher connectivity strength was found after ingesting glucose than with placebo (*p* < 0.01) and fructose (*p* < 0.01), while an increase in connectivity was found in the EC network after fructose ingestion (*p* < 0.05) compared with placebo. Moreover, repeated measure ANOVA revealed significant differences in functional connectivity in the salience network too for the three groups **(B)**. In particular, significantly higher connectivity strength was found after ingesting glucose than with placebo (*p* < 0.05) and fructose compared with placebo (*p* < 0.05). No differences in connectivity were found between glucose and fructose ingestion. Mean and standard errors are reported. Significant levels are reported using the conventional*. *Z*-stat values are shown in the color bar. The results are given by repeated measures permutation-based non-parametric test (randomize, FSL tool) approach, correcting for multiple comparisons by threshold-free cluster enhancement (27). *p*-Values <0.05 were considered as significant, **p < 0.01.

Group analyses of frame wise displacement found no significant effect of motion between the visits. Cross value correlations are for the EF network: *r* = 0.3, *p* < 0.01 and for the SN *r* = 0.4, *p* < 0.01. Repeated measure ANOVA revealed a significant main effect in functional connectivity in the fronto-parietal network (FPN) network [*F*(2, 11) = 13.69, *p* < 0.001] (Figure 3A). Subsequent *post hoc* testing showed significantly higher connectivity strength after ingesting glucose than with placebo (*p* < 0.05) or fructose (*p* < 0.05), while an increase in connectivity in the FPN network was found after fructose ingestion compared with placebo (*p* < 0.01). Moreover, repeated measure ANOVA also revealed a significant main effect of treatment in functional connectivity in the SN network [*F*(2, 11) = 6.117, *p* < 0.05] (Figure 3B). In particular, significantly higher connectivity strength than with placebo was found after ingesting glucose (*p* < 0.05) or fructose (*p* < 0.05). No differences in connectivity were found between glucose and fructose ingestion.

### Cross-Modalities Correlations Results

Significant correlations were found between the N-back results (*p* < 0.05) and resting state connectivity values, both for FPN (*p* < 0.05) and SN (*p* < 0.05). For the SN, significant correlations were found (*p* < 0.05) between the Go/-No-Go results and the resting state connectivity values. The results are corrected for false discovery rate multiple comparison corrections. Results are displayed in Table 1.

## Discussion

The present study performs an extensive assessment of cognition-related brain functional changes after glucose and fructose administration. Although we found no significant differences in behavioral performance during working memory processing and response inhibition, both glucose and fructose decreased activation in frontal areas such as the ACC and dorso-lateral pre-frontal cortex (DLPFC) during working memory processing and response inhibition—especially after fructose intake. The connectivity of these regions as parts of the FPN and SN is in turn increased during glucose and fructose ingestion.

Our first group of results relate to the absence of differences in task performance during working memory processing and response inhibition after glucose and fructose intake compared with placebo. The absence of changes in performance after fructose intake is confirmed by animal studies that found no differences in cognitive/motor performance as measured by object recognition and fear conditioning in rodents (35, 36) and by a recent review (11) that concluded that fructose does not induce cognitive deficits. Published reports on behavioral differences after glucose administration are inconsistent with respect. Although a previous study (37, 38) reported improvements in object recognition and word-recall performance after glucose intake, other authors have found no differences in cognitive performance (39–41). In the present study, we confirm the absence of changes at the behavioral level after sugar administration. From our perspective, this is still an open field of research and our results with this small sample size are not definitive. Although fMRI data on small subject numbers are relatively robust (42), behavioral indexes are typically underpowered and could be confounded by many personal attributes that cannot be clearly assigned to the cognition required for adequate task performance (43).

Our second group of results relate to changes at the level of brain function. During working memory processing, decreased activation in the ACC and DPFC was shown after glucose administration. Less activation in the ACC/DPFC and in the sensory cortex was found after fructose administration than after glucose administration.

As previous studies on cognitive functions and induced-training suggested, decreased brain activation during a demanding cognitive load is associated with more efforts to perform a task (44–47). According to this interpretation, our results suggest the subjects show less demanding brain activation during the stimulus-response association task after glucose and fructose intake than with placebo (48, 49). Moreover, our findings are in line with a recent study that concluded that after glucose and fructose intake the participants showed significantly decreased cerebro-spinal fluid relative to placebo, particularly in the ACC, insula, and thalamus compared with Ref. (9).

In comparison with placebo, we found reduced functional activation in the ACC, DPFC, insula, DLPFC, and visual cortex after glucose and fructose administration. No differences between glucose and fructose were found, which was comparable with the results during working memory processing.

Although working memory involves temporary storage and manipulation of the information (50) and response inhibition involves the suppression of actions that are no longer required or inappropriate (51), our results indicate that acute glucose and fructose administration similarly modulates brain activation during these two cognitive processes.

Our third group of findings relates to differences in resting state functional connectivity after fructose and glucose intake. The connectivity within the FPN and the SN is increased during both fructose and glucose intake compared with placebo; this is comparable with the task-induced fMRI findings, but in the opposite direction.

The increase in connectivity after glucose intake has already been reported several times (4, 9, 52, 53), but ours is the first study to demonstrate increased functional connectivity in networks related to cognitive functions after fructose intake.

Our correlation analyses confirm that glucose and fructose intake lead to increased functional connectivity in the FPN and SN and to decreased efforts during working memory and response inhibition tasks.

We finally want to mention, as already suggested in Section “Introduction,” that food intake may play a significant role beside age and gender in multimodal neuroimaging studies (18). The present work can be considered as a starting point for future investigations also outside the brain-gut field, suggesting to assess nutrient intake beside age, and gender changes on brain functional activity, using them for instance as covariates in the fMRI analyses.

### Limitations

Some limitations of our study merit comment. As in previous neuroimaging studies of the brain-gut axis in healthy subjects, our sample size was modest since it is intended to be a pilot study. In addition, the present study focused only on glucose and fructose, while sucrose and other substances could also be investigated. Our results might potentially be influenced by external factors such as daily mood variations not investigated by examination of the health status of the participants. However, it is important to point out/emphasize that the aim of the present study was to investigate changes in brain networks involved in cognitive functions and this was why emotional changes were not studied in detail. In the fMRI analyses, it is important to notice that smoothing may introduce spurious local functional connectivity and affect the subsequent conduction of ICA, but we decided to keep the smoothing in order to reduce noise. We also want to stress that while we randomized for the treatment assignment order, and for the sequence of stimuli during the tasks, we did not randomize for the fMRI sequences ordering. We therefore suggest future investigations to randomize also for the fMRI tasks ordering, to control for ordering effects.

To conclude, the results of the present work suggest the presence of two partially overlapping neural pathways related to cognitive functions after glucose and fructose ingestion. The working memory and the response inhibition pathways showed that glucose and fructose decrease activation and increase connectivity strengths in regions in the FPN and the SN. These results are to be considered as part of a preliminary and exploratory investigation of sugar effects on cognitive functions. Our findings suggest that future studies on diet-induced manipulations are plausible and efficient for pathologies affecting the cognitive dimension.

## Data Availability Statement

The datasets generated during and/or analyzed during the current study are available from the corresponding author on reasonable request.

## Author Contributions

BW, AM-G, CB, and SB conceived and designed the experiments. BW, AM-G, and KJ performed the experiments. DZ, AD, CS, AS, SH, CLR, and JD data analyses. DZ, CB, and SB wrote the paper.

## Conflict of Interest Statement

The authors declare that the research was conducted in the absence of any commercial or financial relationships that could be construed as a potential conflict of interest.
